# Irisin attenuates ventilator‐induced diaphragmatic dysfunction by inhibiting endoplasmic reticulum stress through activation of AMPK

**DOI:** 10.1111/jcmm.18259

**Published:** 2024-04-27

**Authors:** Jumei Zhang, Rui Tu, Fasheng Guan, Jianguo Feng, Jing Jia, Jun Zhou, Xiaobin Wang, Li Liu

**Affiliations:** ^1^ Department of Anesthesiology The Affiliated Hospital of Southwest Medical University Luzhou China; ^2^ Anesthesiology and Critical Care Medicine Key Laboratory of Luzhou Southwest Medical University Luzhou China

**Keywords:** AMPK, diaphragmatic dysfunction, endoplasmic reticulum stress, irisin, mechanical ventilation, oxidative stress

## Abstract

Mechanical ventilation (MV) is an essential life‐saving technique, but prolonged MV can cause significant diaphragmatic dysfunction due to atrophy and decreased contractility of the diaphragm fibres, called ventilator‐induced diaphragmatic dysfunction (VIDD). It is not clear about the mechanism of occurrence and prevention measures of VIDD. Irisin is a newly discovered muscle factor that regulates energy metabolism. Studies have shown that irisin can exhibit protective effects by downregulating endoplasmic reticulum (ER) stress in a variety of diseases; whether irisin plays a protective role in VIDD has not been reported. Sprague–Dawley rats were mechanically ventilated to construct a VIDD model, and intervention was performed by intravenous administration of irisin. Diaphragm contractility, degree of atrophy, cross‐sectional areas (CSAs), ER stress markers, AMPK protein expression, oxidative stress indicators and apoptotic cell levels were measured at the end of the experiment.Our findings showed that as the duration of ventilation increased, the more severe the VIDD was, the degree of ER stress increased, and the expression of irisin decreased.ER stress may be one of the causes of VIDD. Intervention with irisin ameliorated VIDD by reducing the degree of ER stress, attenuating oxidative stress, and decreasing the apoptotic index. MV decreases the expression of phosphorylated AMPK in the diaphragm, whereas the use of irisin increases the expression of phosphorylated AMPK. Irisin may exert its protective effect by activating the phosphorylated AMPK pathway.

## 1 INTRODUCTION

Mechanical Ventilation (MV) is the most common respiratory support technique in the intensive care unit (ICU) and is widely used in clinical practice, with more than 15 million patients worldwide each year using MV to provide adequate pulmonary ventilation during surgical procedures and critical illnesses.^1^, ^2^ However, MV is a double‐edged sword; prolonged MV can cause diaphragm fibre atrophy and diaphragm weakness, leading to diaphragmatic contractile dysfunction, called ventilator‐induced diaphragm dysfunction (VIDD). VIDD not only has a great adverse impact on patient survival and prognosis but is a major clinical cause of difficulty in weaning from MV, which can result in a large medical burden. More than 50% of mechanically ventilated patients rapidly develop VIDD within 24 h of tracheal intubation.^3^, ^4^ Currently, there is a lack of appropriate clinical measures to prevent and treat VIDD. Therefore, a detailed understanding of the mechanisms of VIDD and preventive measures is essential. Although the molecular mechanisms leading to VIDD are still unclear, oxidative stress, mitochondrial dysfunction, and activation of the protein hydrolysis system have been identified as playing a major role.^5^, ^6^, ^7^ It is well known that protein hydrolysis mediated by the ubiquitin‐proteasome system (UPS) plays a dominant role in skeletal muscle atrophy. Muscle atrophy F‐box (MAFbx)/Atrogin‐1 and muscle RING finger‐1 (MuRF‐1) are two muscle‐specific E3 ubiquitin ligases and are highly expressed in skeletal muscle under a variety of atrophy conditions.^8^, ^9^

The endoplasmic reticulum (ER) is a membrane‐bound organelle that is the primary site of protein synthesis and transport, protein folding, calcium storage and lipid metabolism. ER stress is manifested by aggregation of misfolded and unfolded proteins and disturbances in Ca^2+^ homeostasis when ER function is perturbed by the cellular environment.^10^, ^11^ Subsequently, the unfolded protein response (UPR) is activated to reduce the accumulation of misfolded proteins, which is initiated by three ER transmembrane sensors: protein kinase RNA‐like endoplasmic reticulum kinase (PERK), inositol‐requiring enzyme 1 (IRE1) and activating transcription factor 6 (ATF6).^12^, ^13^, ^14^ ER stress induces a protective effect by activating endoplasmic reticulum molecular chaperones, such as glucose‐regulated protein 94 kD (GRP94) and glucose‐regulated protein 78 kD (GRP78), which assist and participate in the correct folding of proteins.^15^, ^16^, ^17^, ^18^ GRP78 binds to the ER luminal portion of the three receptors, PERK, IREl and ATF6, during the non‐stressed state, and the receptor proteins are inactive in this case. When unfolded proteins accumulate in the ER, GRP78, which has a strong binding capacity to unfolded proteins, dissociates and is released into the ER lumen to perform protein folding functions.^19^ The expression of GRP78 is significantly up‐regulated under ER stress conditions, and thus GRP78 can be used as a marker of ER stress and UPR activation.^20^ CCAAT enhancer binding protein (C/EBP) homologous protein (CHOP) is a downstream transcription factor that is activated at multiple levels during ER stress.^21^ When severe irreversible ER stress is present, downstream activation by the caspase‐12‐mediated apoptotic pathway occurs. Including activation of the caspase family and activation of apoptosis‐associated factors such as Bcl‐2 and Bax.^22^, ^23^ Jiao et al.^24^ recently revealed that activation of ER stress is involved in diaphragm contractile dysfunction during sepsis. ER stress has recently been reported to be present in VIDD and can cause diaphragm atrophy and weakness.^25^ The endoplasmic reticulum has an important role in muscle contractile activity as a processing plant for intracellular proteins and a reservoir for calcium ions. ER stress can also cause impaired muscle protein synthesis, leading to muscle atrophy. There is a strong link between ER stress and muscle atrophy and dysfunction.^12^, ^26^ Therefore, reducing ER stress is an effective strategy to improve VIDD.

Irisin is a newly discovered proliferator‐activated receptor‐γ coactivator‐1α (PGC‐1α)‐dependent myokine that is produced primarily in exercising muscles.^27^ Irisin has anti‐inflammatory and antioxidant effects and may play an ameliorating role in acute pancreatitis, myocardial ischemia–reperfusion injury and liver fibrosis by attenuating ER stress.^28^, ^29^ However, studies of irisin in VIDD have not been reported. AMPK, a cellular energy sensor, is strongly activated in skeletal muscle by repetitive muscle contraction and exercise in rodents and humans.^30^, ^31^ AMPK is thought to stimulate mitochondrial biogenesis as well as lipid and glucose metabolism in skeletal muscle.^32^ Activation of the AMPK/SIRT1/PGC‐1α signalling cascade attenuated skeletal muscle atrophy in ageing rats, possibly because AMPK regulates autophagy and ubiquitin proteasome‐mediated catabolism in skeletal muscle.^33^ It was shown that the proportion of slow muscle fibres could be increased by up‐regulating the AMPK signalling pathway, thereby improving the fatigue resistance of skeletal muscle.^34^ In the case of cascade activation, it enhances the protective effect of cells under stressful conditions. Many studies have reported that irisin can upregulate AMPK to regulate ER stress and exert a protective effect in different disease models.^35^, ^36^, ^37^ There is a close link between AMPK and irisin. Firstly, during muscle exercise, AMPK is activated and irisin synthesis and secretion are stimulated simultaneously.^38^ Secondly, irisin has been shown to induce AMPK phosphorylation and activation in vitro.^39^ Another research group has shown that skeletal muscle AMPK is necessary to maintain basal irisin expression by comparing AMPK knockout mice to wild‐type mice and observing a dramatic decrease in irisin expression in the first group of mice.^40^ Muscle secretion of irisin is dependent on AMPK signalling. In turn, irisin has an autocrine effect, increasing the phosphorylation pathway of AMPK and thus improving muscle metabolism and function.^39^, ^41^ Recent studies have noted that ER stress can cause VIDD. But whether irisin and AMPK are involved in the process of VIDD is unclear. In the present study, we found for the first time that irisin expression decreased progressively in the diaphragm with increasing ventilation time. Therefore, the present study hypothesised that administration of exogenous irisin could ameliorate VIDD by down‐regulating ER stress and attenuating oxidative stress and resulting apoptosis through activation of AMPK.

## 2 MATERIALS AND METHODS

### 2.1 Experimental Animals

Adult male Sprague–Dawley rats weighing 280–320 g were purchased from Huafukang Biotechnology Co. (Beijing, China). The animals were kept in sterile laminar flow rat cages (temperature: 25 ± 2°C, relative humidity: 50% ± 5%), daytime alternation, animals can freely eat and water. All of our experimental procedures are performed in accordance with the Institutional Guidelines for the Use and Care of Laboratory Animals. Animal experiments were approved by the Ethics Committee of the Animal Experiment Center of Southwest Medical University (Animal Ethics Review No: swmu20230072).

### 2.2 Experimental design

#### 2.2.1 Experiment 1

To demonstrate whether prolonged ventilation in rats activates ER stress and to investigate the relationship between ER stress and diaphragm dysfunction as the duration of ventilation increases. Sprague–Dawley rats were randomly assigned to one of five experimental groups: group 1, control group, rats were anaesthetized (*n* = 6); group 2, MV6h, animals receiving 6 h of MV(*n* = 6); group 3, MV12h, animals receiving 12 h of MV(*n* = 6); group 4, MV18h, animals receiving18h of MV(*n* = 6); group 5, MV24h, animals receiving 24 h of MV(*n* = 6).

#### 2.2.2 Experiment 2

To demonstrate whether irisin can ameliorate MV diaphragm dysfunction through AMPK downregulation of ER stress. Sprague–Dawley rats were randomly assigned to one of three experimental groups: group 1, control group, rats were anaesthetised (*n* = 6); group 2, MV12h, animals receiving 12 h MV (*n* = 6); group 3, Irisin + MV12h, animals receiving 12 h MV and irisin treatment (*n* = 6).

### 2.3 MV treatment

Rats were acutely anaesthetized by intraperitoneal injection of 60 mg/kg of sodium pentobarbital. After successful anaesthesia, the animal was placed on a temperature‐controlled heating blanket and secured. Then, the neck skin was prepared (skin preparation and disinfection), and the free trachea was dissected aseptically. A 0.3‐cm incision was then cut with ophthalmic scissors and an endotracheal tube was inserted, and a small animal ventilator (Rivard, China) in volume‐controlled mode was connected. The ventilator parameters were set to a tidal volume (VT) setting of 8 mL/kg body weight, a PEEP of 0 cm H_2_O and a respiratory rate (RR) of 60–75 bpm, which could be adjusted appropriately for the rats. 24GY indwelling needle (heparin pre‐filled) was placed in the tail vein and pumped continuously with 2 mL/kg/h of saline and 10 mg/kg/h of sodium pentobarbital. During the experiment, the body temperature was maintained at 37 ± 0.5°C by using a heating blanket. During the experiment, regular care was provided: turning, bladder massage and suctioning. Irisin (PEPROTECH, New Jersey, USA) was injected at 10 μL/kg from the tail vein 10 min before MV.

### 2.4 Muscle fibre cross‐sectional areas (CSAs)

Fragments of each group of diaphragms were fixed in 10% paraformaldehyde. The tissue samples were then subjected to conventional paraffin embedding to make continuous paraffin‐embedded sections of approximately 8 μm thickness. The main steps of staining: the sections were first dewaxed to water, stained with haematoxylin and then fractionated using hydrochloric acid ethanol, counterblue in warm water and placed in 85% ethanol for 5 min and stained with eosin, again dehydrated by gradient ethanol and sealed transparently. Histopathological changes of the diaphragm were observed under light microscopy. Then, the CSAs of diaphragm cells were measured and statistically analysed using Image‐Pro Plus 6.0 software.

### 2.5 Non‐invasive compound muscle action potential (CMAP) detection

Refer to our team's previous modelling methods.^42^ Diaphragmatic CMAP was recorded using a bioinformatics medical signal acquisition system. CMAP is the sum of action potentials of muscle fibres recorded from the muscles it innervates (diaphragm) that occur almost synchronously after stimulation of the phrenic nerve. Referring to previous studies by our team, each electrode is inserted in turn into the designated position. The stimulator was then connected to the stimulating electrode, and the stimulation was single stimulation and positive voltage stimulation. The stimulation intensity was 15 V, the wave width was 1 ms, and the delay was 1 ms. There was a 30‐s interval between stimulations, and we repeated the stimulation three times consecutively for each rat to obtain the average response.

### 2.6 Measurement of diaphragm frequency‐contraction curve and fatigue index

Ribbed muscle strips approximately 3 mm wide and 1 cm long were placed in Krebs‐Hensleit solution, equilibrated with 95% O_2_‐5% CO_2_ gas and maintained at 37°C and pH 7.4. One end of the muscle strip was attached to the muscle force receptor with a suture and one end was fixed. Use the maximum stimulation voltage (15 V) to determine the optimal contraction length (Lo). The muscle was then continuously stimulated at 20, 40, 60, 80, 100 and 120 Hz for 600 ms with a 1‐min interval between each stimulation to determine the frequency contraction curve relationship and to calculate the contraction force in terms of physiological cross‐sectional area. The length and weight of the muscle strips were measured at the end of the experiment. Muscle density was calculated as 1.06 (g/cm^3^), and the cross‐sectional area of the strips was calculated as cm^2^ = weight/density/length, and the contraction force of the strips was normalised by the cross‐sectional area: force/cross‐sectional area (g/cm^2^). The muscles were continuously stimulated for 2 min at 50 Hz using a pulsed square wave (intensity 15 V, wave width 0.5 ms, delay 20 ms). To determine the fatigue index, the initial force magnitude was divided by the force magnitude generated after 2 min of stimulation.

### 2.7 Western blot assay

The expression levels of ERS markers, diaphragm atrophy proteins, and apoptosis‐related proteins were detected by Western blot. The diaphragm tissue was first homogenised and the supernatant was taken. Protein concentrations were measured using a BCA kit (Beyotime, China). Protein loading buffer was added to each sample, then boiled for 10 min, centrifuged at 4°C for 10 min and stored. The proteins were separated after electrophoresis with sodium dodecyl sulfate polyacrylamide gel at constant pressure 120 V for 1.5 h. The polyvinylidene difluoride membranes received the proteins under a steady current of 220 mA. Following this, the membranes were sealed with skimmed milk and allowed to incubate at room temperature for 1 h. After that, they were placed in contact with primary antibodies and left for the night in a cold temperature of 4°C. Major primary antibodies include: MuRF‐1(1∶1000, Santa Cruz, USA, SC‐398608), Atrogin‐1/MAFbx(1∶1000, Santa Cruz, USA, SC‐166806), GRP78(1∶1000, Abcam, UK, ab108615), CHOP(1∶1000, Cell Signalling Technology, USA, CST2895S), GRP94(1∶1000, Cell Signalling Technology, USA, CST20292S), Bax (1:1000, Affinity, China, AF0120), Bcl‐2(1:1000, Affinity, China, AF6138), Irisin(1:1000, Abcam, UK, ab174833), AMPK(1:1000, Affinity, China, AF6422), Phospho‐AMPK(1:1000, Affinity, China, AF3422), GAPDH(1:5000, Santa Cruz, USA, SC‐32233). Finally, after an overnight incubation, further incubation with secondary antibody for 1 h at room temperature was followed by three washes with PBS. The final development was performed with developing solution (Biotec Biotech, Shanghai, China).

### 2.8 Real‐time quantitative polymerase chain reaction(qPCR)

To obtain RNA from each diaphragm tissue group, we used TRIzol reagent (Life Technologies, Carlsbad, CA) for isolation. The reverse transcription of the isolated RNA into cDNA was carried out using the qPCR kit (Vazyme, China). The experiments were performed according to the kit instructions, and the PCR reaction solution was prepared with a total system of 25 μL (Prolight Bio, China). To perform the PCR reaction, we set the temperature at 95°C for 30 s followed by 40 cycles of 95°C for 5 s and 60°C for 30 s. Amplification specificity was verified by dissociating the product to generate a melting curve. As a reference gene, we utilised GAPDH. You can find the primer sequences for each gene listed in Table 1.

**TABLE 1:** Sequences of the PCR primers for amplification of expressed genes.

Primers	Primer sequences
MuRF‐1	F: CGGACGGAAATGCTATGGAGAACC
	R: GGATTGGCAGCCTGGAAGATGTC
Atrogin‐1/MAFbx	F: TGGATGAGAAAAGCGGCACCTTC
	R: TCTCTTCTTGGCTGCAACATCGTAG
CHOP	F: CGCATGAAGGAGAAGGAGCA
	R: TGTGGTCTCTACCTCCCTGG
GRP94	F: CCTGCTGACCTTCGGGTTTGTG
	R: CAACCTTCATCGTCTGTCCGTGAG
GRP78	F: CTGACAACGACAAGACCCCA
	R: CTCCGATTGGTGAACTCGCT
Bax	F: AGGACGCATCCACCAAGAAG
	R: CATCCTCTGCAGCTCCATGT
Bcl‐2	F: GGGATGACTTCTCTCGTCGC
	R: CACCACCGTGGCAAAGC
Irisin	F: CCTTGGCACAGGACTCACAT
	R: TTCTTGTCCCTCGAAGCCAC
GAPDH	F: GGTTGTCTCCTGCGACTTCA
	R: CCCTGTTGCTGTAGCCGTAT

### 2.9 TUNEL staining of diaphragm muscle tissue

We used TUNEL staining to detect apoptotic cells in the diaphragm tissue. The TUNEL detection kit (Beyotime, China) was used to detect apoptotic cells in diaphragm tissue. Paraffin sections were first made in the conventional way and then washed with gradient ethanol once each for 3 min. We added a mixture of TdT and DIG‐d‐UTP to prepare the TUNEL reaction mix, incubated for 2 h at 4°C, and then washed with PBS for three rounds. Add the sealing solution (40 μL) to the slices and seal the slices for 30 min at room temperature. Add rabbit anti‐human HMGN2 monoclonal primary antibody (Cell Signalling Technology, USA) and incubate at 37°C for 40 min. Then wash with PBS three times. Goat anti‐rabbit SABC‐FITC secondary antibody (Cell Signalling Technology, USA) was added, and the sections were placed in a wet box and reacted at 37°C for 40 min. Then, the sections were washed three times with PBS. Anti‐fluorescence quenching blocking buffer was then added dropwise to the sections, observed under a confocal microscope and photographed. There were TUNEL‐positive signals in the nuclei, whereas DAPI‐stained nuclei were blue, while those from apoptotic cells were red.

### 2.10 Measurement of serum irisin and diaphragm ROS, MDA content and GSH‐Px activity

After modelling is complete, blood is collected from the tip of the heart and stored in an anticoagulation tube for 30 min. The blood was centrifuged at 3000 × *g* for 10 min, and the supernatant was collected. Finally, serum irisin concentration was determined using the rat irisin ELISA kit (MEIMIAN, China) according to the kit instructions. Fresh diaphragm tissue from each group was collected, cut and placed in pre‐cooled RIPA lysis buffer (Beyotime, China). After homogenising the samples from each group, the supernatant was collected by aspiration following centrifugation at 12,000 rpm for 15 min at a temperature of 4°C. Malondialdehyde (MDA) glutathione peroxidase activity (GSH‐PX) in the diaphragm was measured with the MDA assay kit and GSH‐PX assay kit (Beyotime, China), according to the manufacturer's instructions. The amount of reactive oxygen species (ROS) was measured with the tissue ROS assay kit (Biorab, China).

### 2.11 Analysis of data

GraphPad.Prism.9.0 software was used for statistical analysis, and Image‐Pro Plus was used to process and analyse the images. Quantitative variables conforming to a normal distribution are expressed as means ± standard deviation (SD) throughout this study. Differences between groups were determined by one‐way analysis of variance (ANOVA) followed by post‐hoc testing by Tukey's test. *p* < 0.05 was considered statistically significant (two‐tailed). The number of animals per group required to identify significant differences in major parameters was determined based on previous experience with the same mode.

## 3 RESULTS

### 3.1 Diaphragm dysfunction worsens with prolonged ventilation time

**FIGURE 1:**
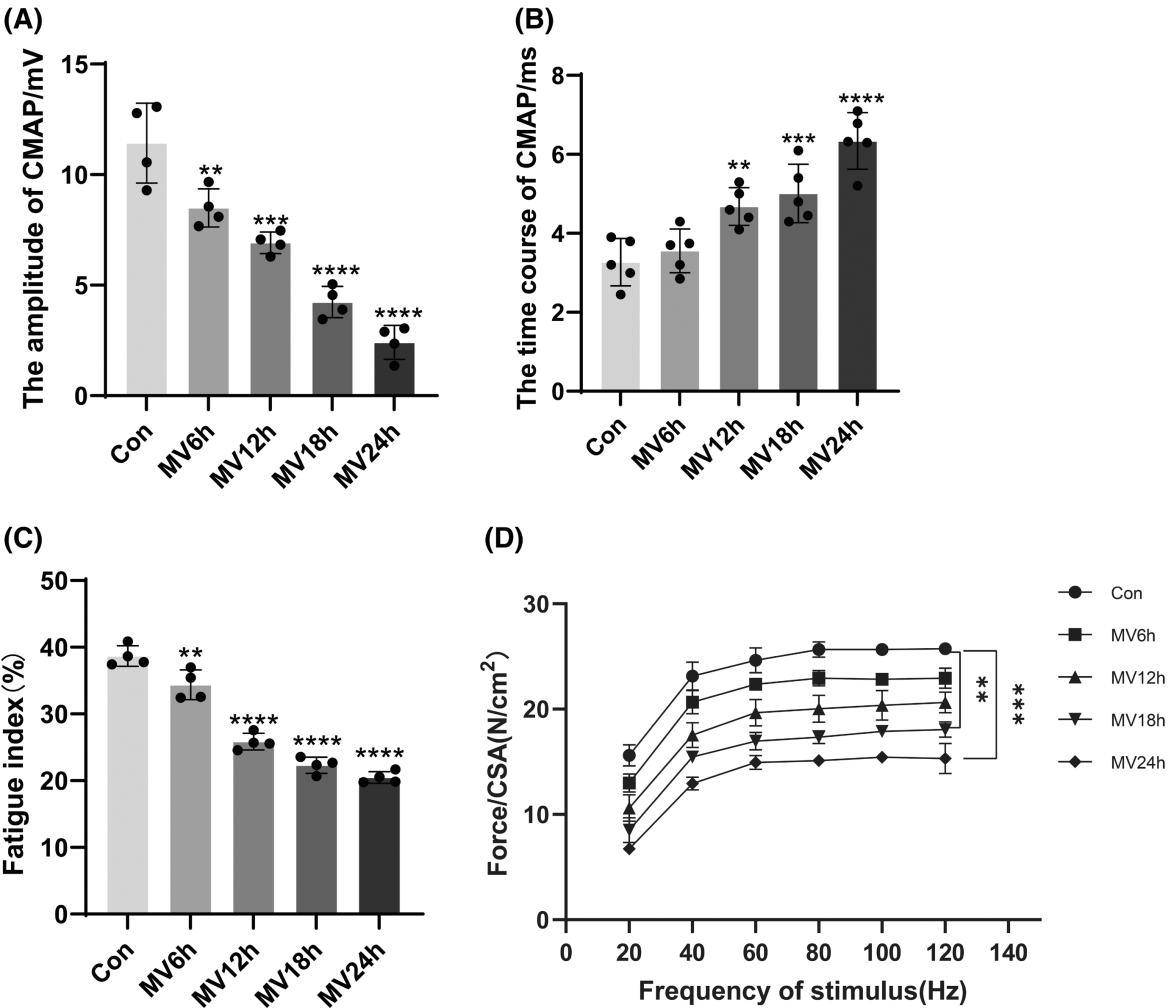
Diaphragmatic functional indicators. (A) Each group's CMAP amplitude; (B) The CMAP time course of each group; (C) Diaphragm fatigue index for each group; (D) Force‐frequency curve of diaphragm muscle. The measured data are expressed as mean ± SD. The experiment was repeated not less than thrice. **p < 0.01 versus with control group; ***p < 0.001 versus with control group; ****p < 0.0001 versus with control group. MV, mechanical ventilation; CMAP, compound muscle action potential; CSA, cross‐sectional area.

Consistent with other reports in the literature, prolonged MV can cause diaphragm dysfunction.^43^, ^44^ CMAP amplitude decreased gradually with the prolongation of ventilation time in each group, and the time course of CMAP was gradually prolonged and was most obvious in MV24h group (Figure 1A,B). With prolonged ventilation, the ratio of the force generated after 2 min of continuous stimulation to the force generated at the beginning of the stimulation became smaller and smaller, that is, the fatigue index, and the diaphragm's resistance to fatigue gradually diminished (Figure 1C). At the same stimulation frequency, the contraction force of the diaphragm was greatest in the control group and became less and less with the prolongation of the ventilation time (Figure 1D).

### 3.2 Diaphragmatic atrophy increases with prolonged ventilation time

**FIGURE 2:**
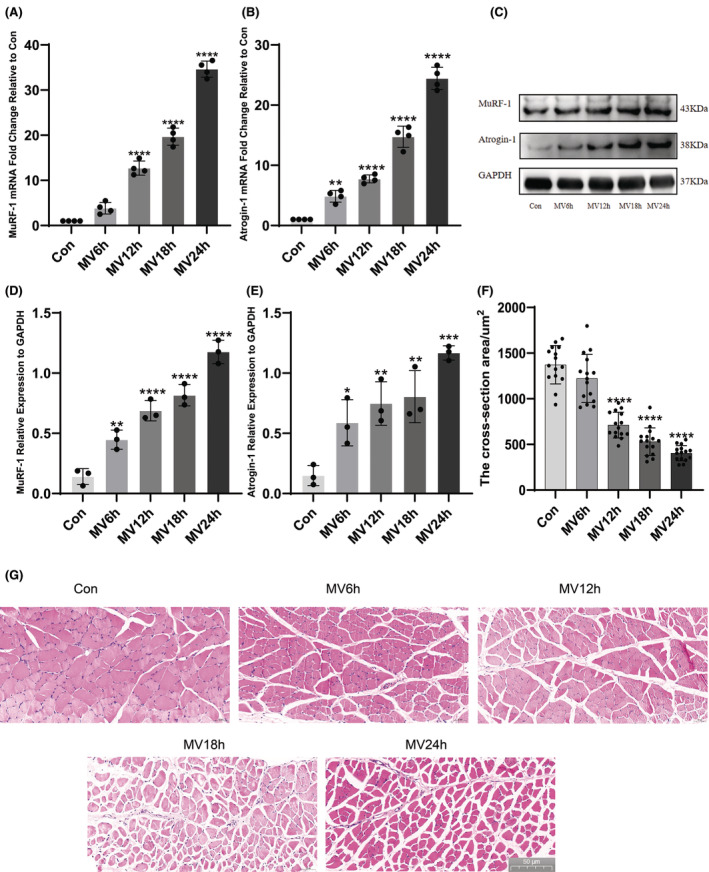
Protein and mRNA expression of Atrogin‐1/MAFbx and MuRF1 in each group and H&E staining of the diaphragm. (A) The expression levels of MuRF‐1 mRNA were analysed in each group; (B) The expression levels of Atrogin‐1/MAFbx mRNA were analysed in each group; (C) Protein bands of Atrogin‐1/MAFbx and MuRF‐1 by western blot; (D) Protein expression of MuRF‐1 in each group and (E) Protein expression of Atrogin‐1/MAFbx in each group; (F) Diaphragm CSA statistics for each group; (G) Representative graphs of haematoxylin and eosin staining for each group. The measured data are expressed as mean ± SD. The experiment was repeated no less than thrice. *p < 0.05 versus with control group; **p < 0.01 versus with control group; ***p < 0.001 versus with control group; ****p < 0.0001 versus with control group. MV, mechanical ventilation; CSA, cross‐sectional area.

The ubiquitin proteasome pathway is the predominant pathway of muscle atrophy. MuRF‐1 and Atrogin‐1/MAFbx are among the key muscle‐specific E3 ligases. We usually use the expression of MuRF‐1 and Atrogin‐1/MAFbx to respond to the degree of muscle atrophy. The mRNA levels of the muscle‐specific E3 ligases MuRF‐1 and Atrogin1 increased with increasing MV time, which was most pronounced in the MV24h group (Figure 2A,B). The expression of MuRF‐1 and Atrogin‐1/MAFbx protein levels also gradually increased with the prolongation of ventilation time (Figure 2C–E). MuRF‐1 and Atrogin‐1/MAFbx are two genes that are closely related to muscle protein hydrolysis. Haematoxylin and eosin staining showed that the cross‐sectional area of diaphragm fibres progressively decreased and the space between muscle fibres became larger with prolonged ventilation in the mechanically ventilated group compared with the control group (Figure 2G). The cross‐sectional area of diaphragm fibres gradually decreased (Figure 2F).

### 3.3 ER stress progressively increases with prolonged ventilation time

**FIGURE 3:**
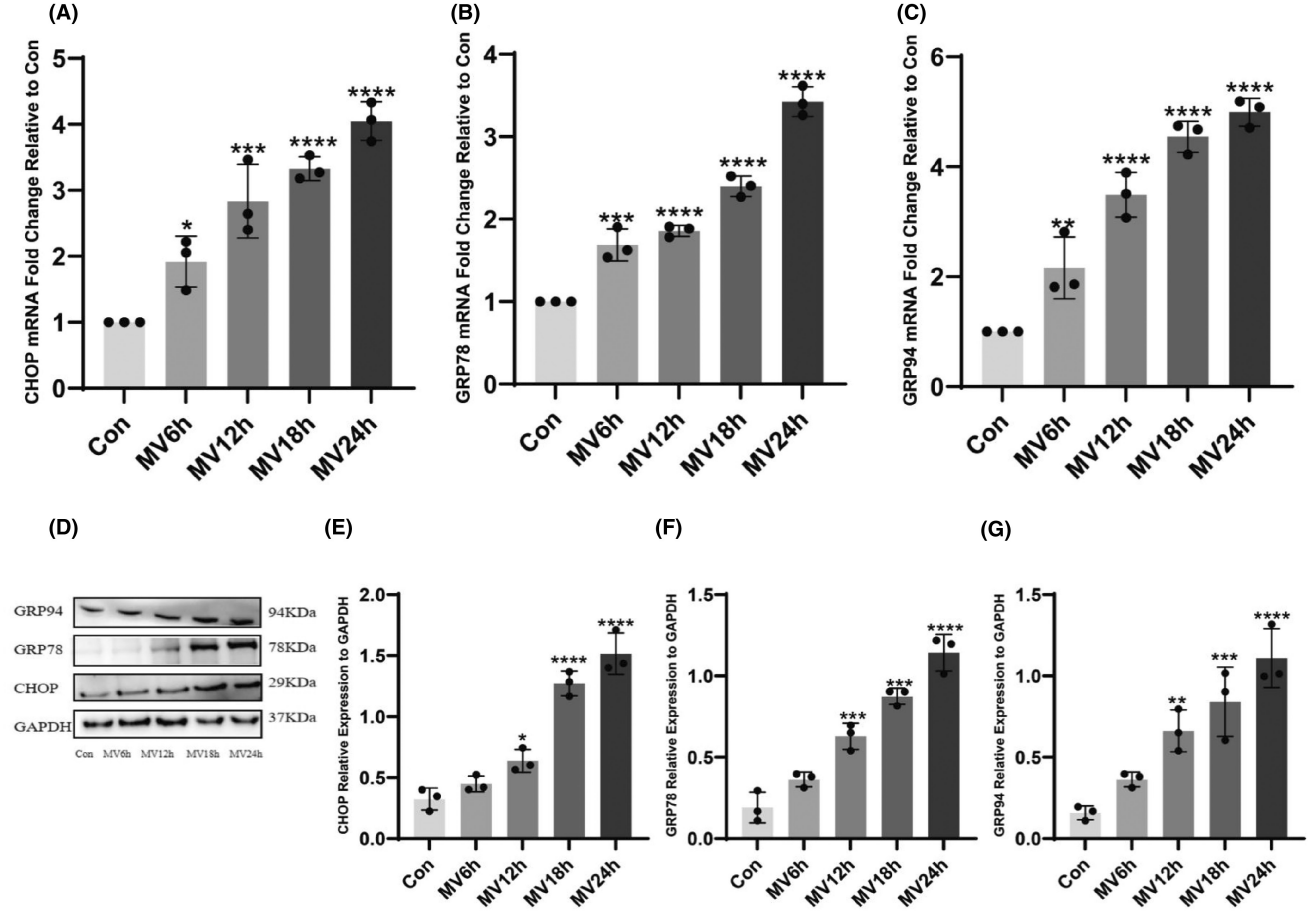
The mRNA and protein expression of ER stress markers. (A) The expression levels of CHOP mRNA were analysed in each group; (B) The expression levels of GRP78 mRNA were analysed in each group; (C) The expression levels of GRP94 mRNA were analysed in each group; (D) Protein bands of CHOP and GRP78, GRP94 by western blot; (E) Protein expression of CHOP in each group; (F) Protein expression of GRP78 in each group; (G) Protein expression of GRP94 in each group. The measured data are expressed as mean ± SD. The experiment was repeated not less than thrice. *p < 0.05 versus with control group; **p < 0.01 versus with control group; ***p < 0.001 versus with control group; ****p < 0.0001 versus with control group. CHOP, C/EBP homologous protein; GRP78, glucose‐regulated protein of 78 kDa; GRP94, glucose‐regulated protein of 94 kDa; MV, mechanical ventilation.

Compared with the control group, the protein and mRNA levels of ER stress markers C/EBP homologous protein (CHOP), glucose‐regulated protein of 78 kDa (grp78) and glucose‐regulated protein of 94 kDa (GRP94) gradually increased with the prolongation of ventilation time, and the degree of ER stress was gradually enhanced, most significantly in the 24‐h group of MV (Figure 3). In the non‐stressed state, GRP78 is bound to the three receptor parts of the endoplasmic reticulum under stress, in which case the receptor proteins are inactive. When proteins are aggregated in the ER and the endoplasmic reticulum is under stress, GRP78 is dissociated and released into the ER lumen to perform protein folding functions.^11^ Thus, GRP78 can be used as a marker of ER stress activation. Activation of ER stress activates the downstream CHOP pathway and causes associated apoptosis. When unfolded proteins are deposited on the endoplasmic reticulum, it activates GRP94 and helps it to fold correctly. Therefore, GRP78, CHOP and GRP94 are usually used as the marker of ER stress.

### 3.4 Irisin expression in the diaphragm of different groups

**FIGURE 4:**
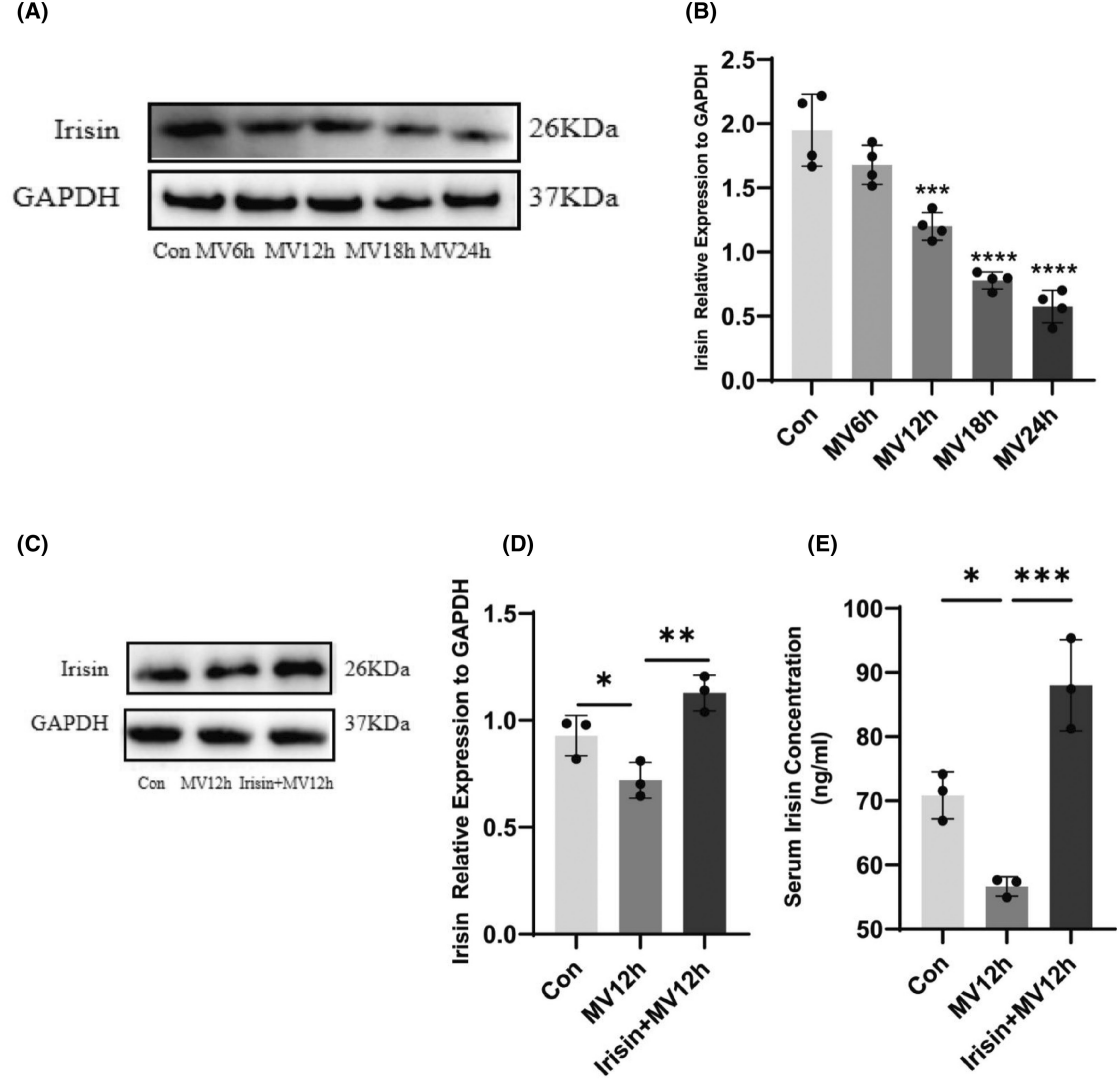
Irisin protein expression in the diaphragm at different ventilation durations. (A) Protein bands of irisin by western blot; (B) The protein expression levels of irisin were evaluated for each group; (C) Irisin protein bands of western blot after intervention; (D) Protein expression of irisin proteins in each group after intervention; (E) Serum irisin protein levels in each group after intervention. The measured data are expressed as mean ± SD. The experiment was repeated not less than three times. *p < 0.05 versus with control group; **p < 0.01 versus with control group; ***p < 0.001 versus with control group; ****p < 0.0001 versus with control group. MV, mechanical ventilation.

In this study, it was found for the first time that irisin protein expression gradually decreased in mechanically ventilated diaphragm with prolonged ventilation time, with the most pronounced decrease in the MV24h group (Figure 4A,B). After intravenous administration of irisin, irisin levels in both serum and diaphragm increased (Figure 4C,D,E).

### 3.5 Irisin improves diaphragmatic dysfunction

**FIGURE 5:**
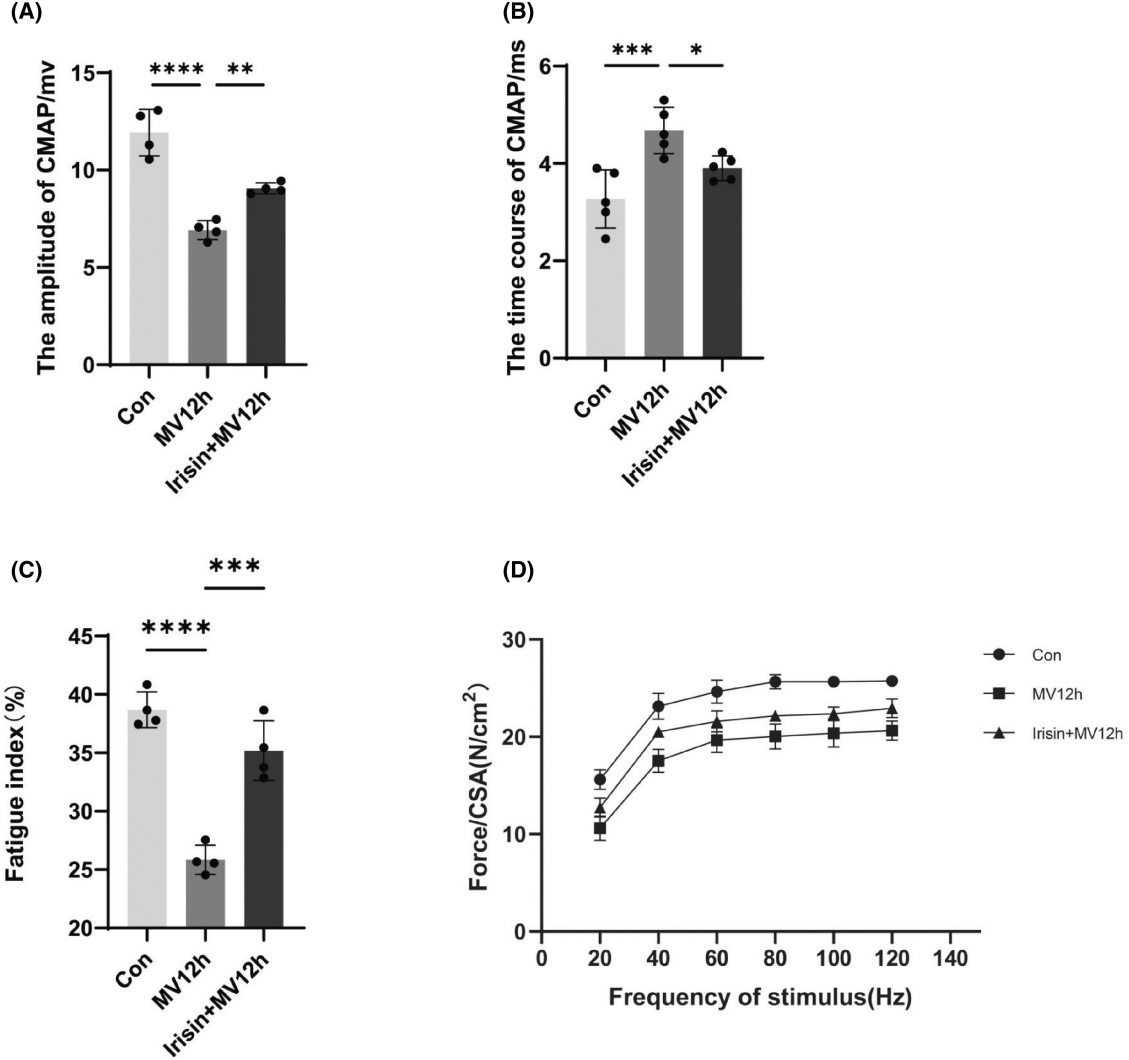
Effect of irisin on diaphragm function. (A) The amplitude of CMAP of each group; (B) The time course of CMAP of each group; (C) Diaphragm fatigue index for each group; (D) Force‐frequency curve of diaphragm muscle. The measured data are expressed as mean ± SD. The experiment was repeated no less than three times. *p < 0.05 versus with MV12h group; **p < 0.01 versus with MV12h group; ***p < 0.001 versus with MV12h group; ****p < 0.0001 versus with MV12h group. MV, mechanical ventilation; CMAP, compound muscle action potential; CSA, cross‐sectional area.

Prolonged MV caused diaphragm dysfunction. After Irisin intervention, MV‐induced diaphragmatic dysfunction was alleviated. This mainly includes the increase of CMAP amplitude and the shortening of CMAP time course (Figure 5A,B). As well as an increase in the fatigue resistance index (Figure 5C). There was an increase in contraction force per cm^2^ at the same stimulation frequency, although there was no statistically significant difference (Figure 5D). These results suggest an improvement in diaphragm function.

### 3.6 Irisin alleviates the degree of diaphragmatic atrophy

**FIGURE 6:**
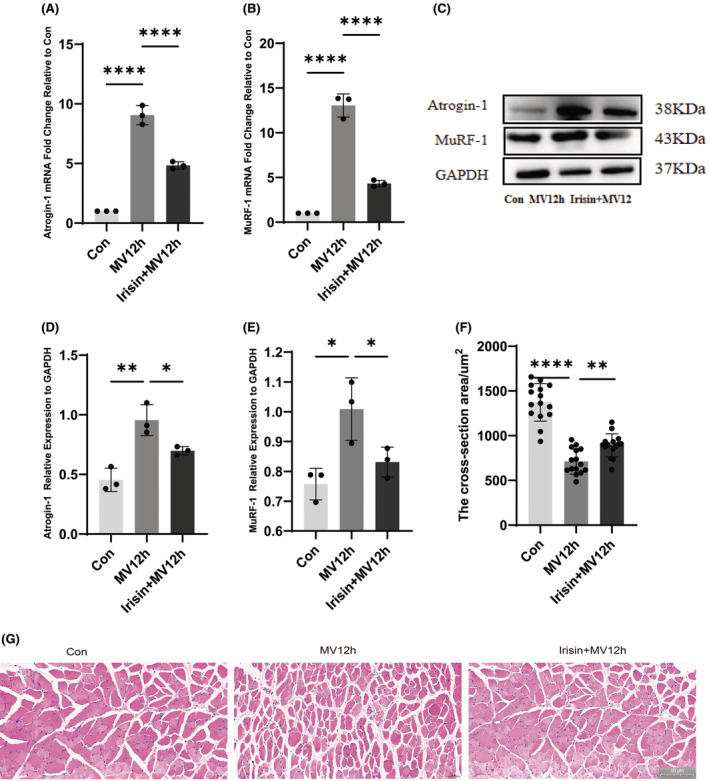
Effect of irisin on diaphragmatic atrophy in MV. (A) The mRNA expression levels of Atrogin‐1/MAFbx in each group; (B) The mRNA expression levels of MuRF‐1 in each group; (C) Protein bands of MuRF‐1 and Atrogin‐1/MAFbx by western blot; (D) The protein expression levels of Atrogin‐1/MAFbx were evaluated for each group; (E) The protein expression levels of MuRF‐1 were evaluated for each group; (F) Results of diaphragm cross‐sectional area statistics; (G) Representative images of each group of H&E staining. The measured data are expressed as the mean ± SD. The experiment was repeated not less than three times. *p < 0.05 versus with MV12h group; **p < 0.01 versus with MV12h group; ****p < 0.0001 versus with MV12h group. MV, mechanical ventilation.

Diaphragm atrophy indicators MuRF‐1 and Atrogin‐1/MAFbx protein and mRNA levels were increased in the 12‐h ventilation group compared with the control group. And the expression of atrophy indicators was lower in the irisin pretreatment group than in the mechanically ventilated group (Figure 6A–E). Diaphragm atrophy was reduced after irisin administration. haematoxylin and eosin staining showed a reduction in the CSA of diaphragm fibres and a slight widening of cell intervals after 12 h of MV. Compared with the MV12h group, the morphology of diaphragm fibres improved, and the CSA became larger after administration of irisin (Figure 6F,G).

### 3.7 Irisin attenuates ER stress in VIDD

**FIGURE 7:**
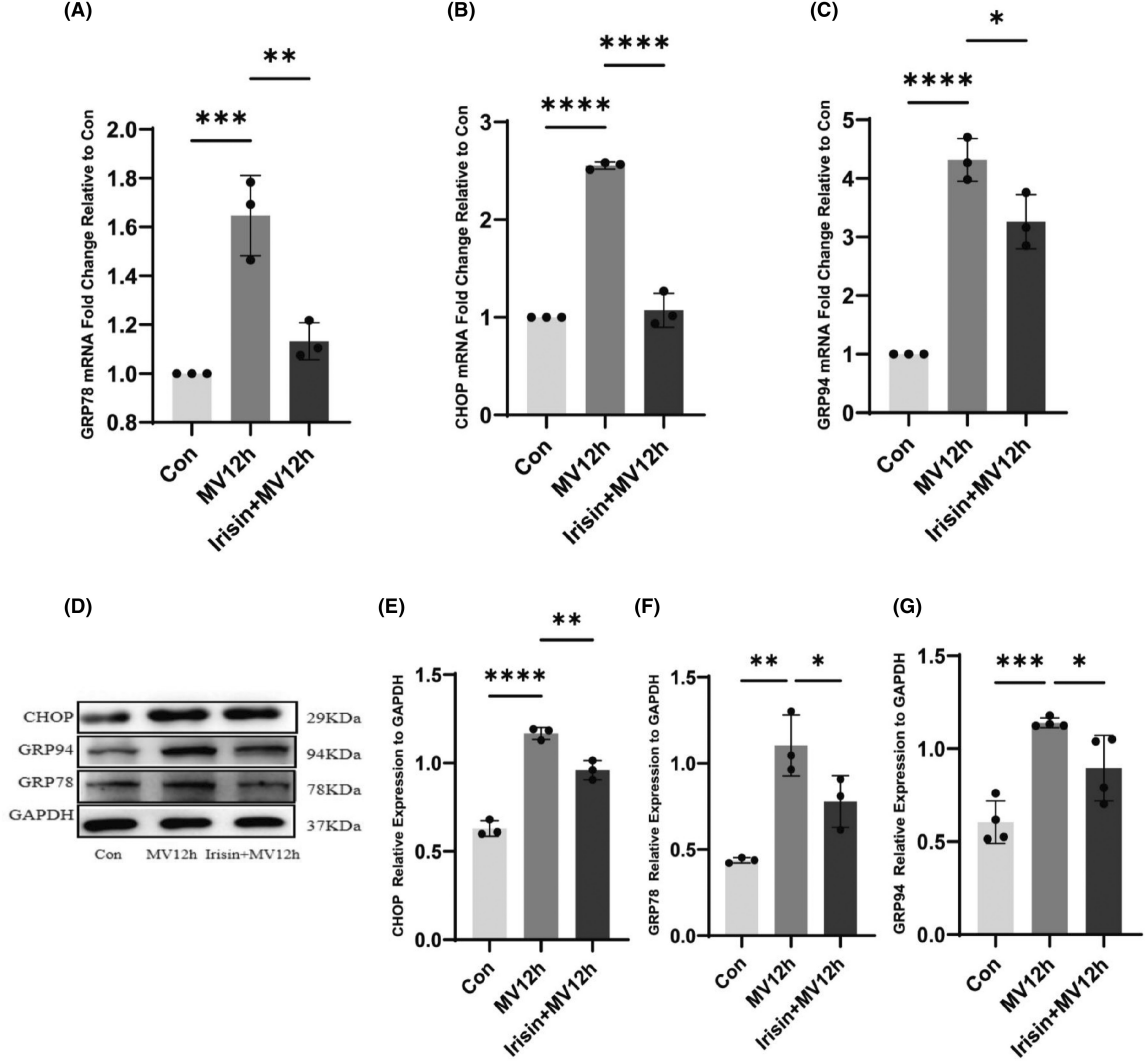
Effect of irisin on ER stress. (A) The expression levels of GRP78 mRNA were analysed in each group; (B) The expression levels of CHOP mRNA were analysed in each group; (C) The expression levels of GRP94 mRNA were analysed in each group; (D) ERS marker protein bands; (E) The protein expression levels of CHOP were evaluated for each group; (F) The protein expression levels of GRP78 were evaluated for each group; (G) The protein expression levels of GRP94 were evaluated for each group. The measured data are expressed as mean ± SD. The experiment was repeated no less than three times. *p < 0.05 versus with MV12h group; **p < 0.01 versus with MV12h group; ***p < 0.001 versus with MV12h group; ****p < 0.0001 versus with MV12h group. CHOP, C/EBP homologous protein; GRP78, glucose‐regulated protein of 78 kDa; GRP94, glucose‐regulated protein of 94 kDa; MV, mechanical ventilation.

Studies have reported that irisin may play a protective role in many diseases by mitigating ER stress. However, its specific mechanism needs further study. To investigate whether irisin can alleviate MV‐related ER stress in the diaphragm, we examined the expression of ER stress‐related proteins in the diaphragm. We examined the expression of ER stress markers using both western blot analysis and qPCR, as shown in Figure 7. MV caused an upregulation of ER stress in the diaphragm, as evidenced by an increase in the ER stress markers CHOP, GRP78 and GRP94 at prote However, its specific mechanism needs further study.in and mRNA levels. The expression levels of ER stress markers decreased after the intervention with irisin, which alleviated the ER stress in the diaphragm caused by MV (Figure 7).

### 3.8 Irisin reduces oxidative stress in the diaphragm

**FIGURE 8:**
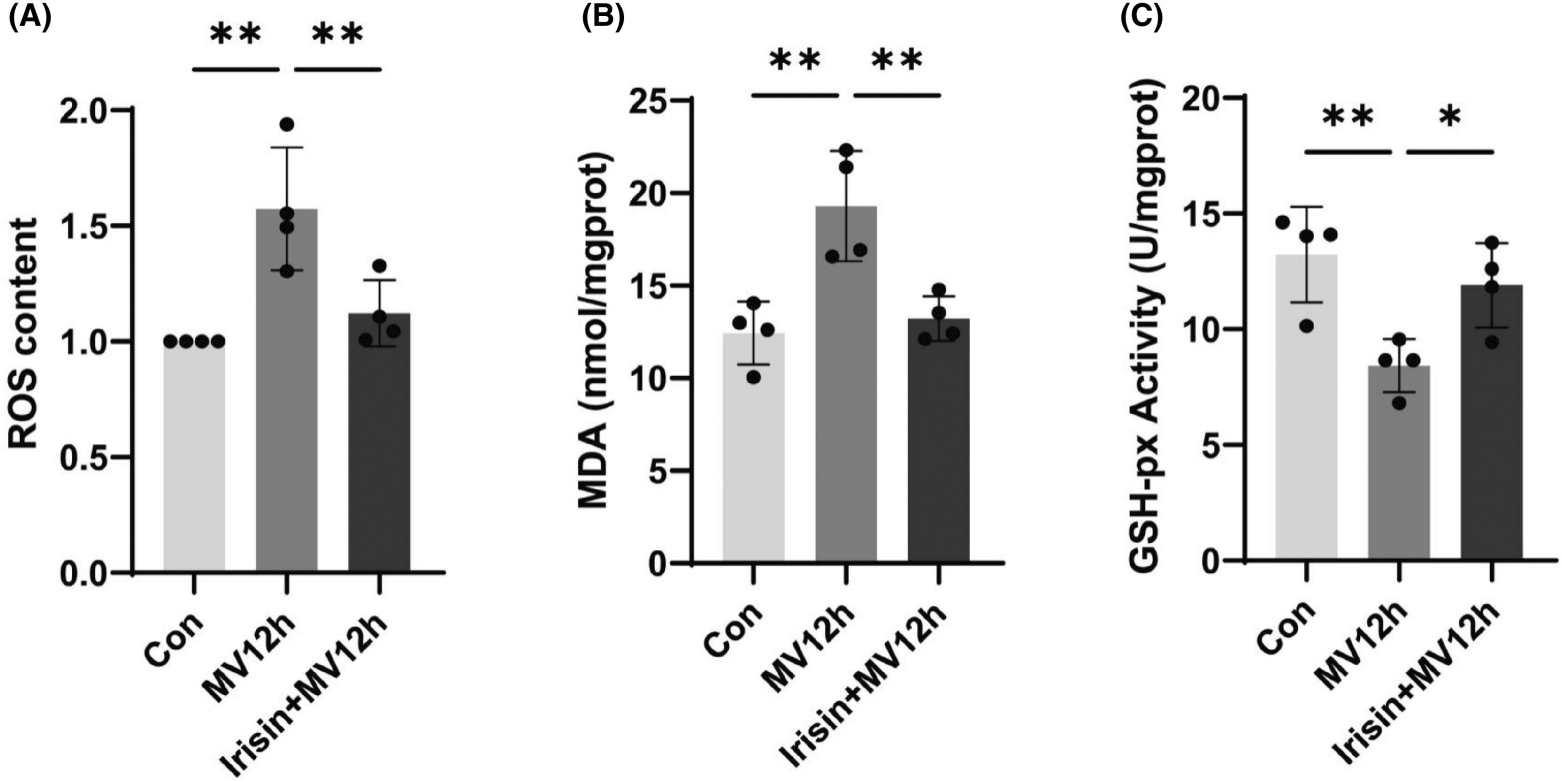
Effect of irisin on oxidative stress in the diaphragm. (A) Diaphragm ROS production in each group; (B) Diaphragm MDA content in each group; (C) GSH‐Px activity. The measured data are expressed as mean ± SD. The experiment was repeated not less than three times. *p < 0.05 versus with MV12h group; **p < 0.01 versus with MV12h group. ROS, reactive oxygen species; MDA, malondialdehyde; GSH‐Px, glutathione peroxidase; MV, mechanical ventilation.

Prolonged MV leads to increased oxidative stress in the diaphragm, and increased oxidative stress activates the protein hydrolase system, leading to increased atrophy of the diaphragm.^1^, ^44^ Oxidative stress and calcium ion disorders are also inextricably linked.^45^ ER stress is usually accompanied by oxidative stress and exacerbates diaphragm cell damage. MV for 12 h caused an increase in diaphragmatic oxidative stress indicators, mainly including ROS, MDA and GSH‐Px, and after the use of irisin, the diaphragmatic oxidative stress induced by MV was reduced. It showed that irisin could reduce oxidative load and oxidative stress damage (Figure 8).

### 3.9 Irisin attenuates apoptosis in the mechanically ventilated diaphragm

**FIGURE 9:**
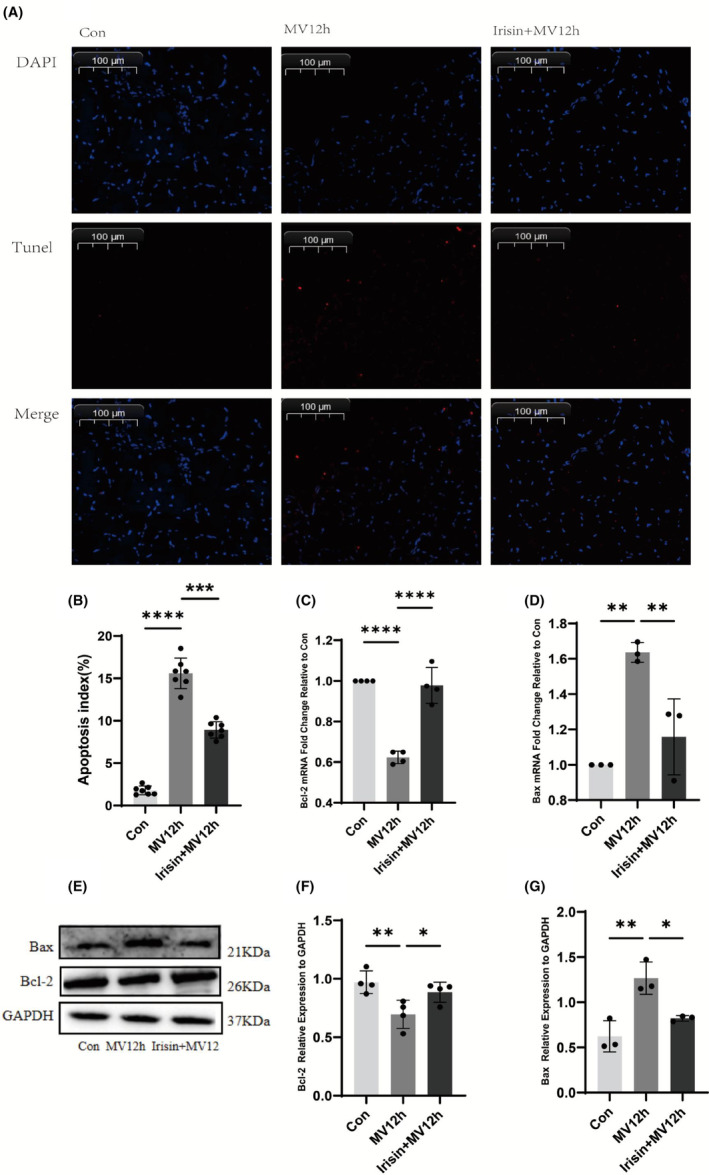
Effect of irisin on diaphragm apoptosis. (A) Representative images of each group of diaphragm in situ TUNEL assay. TUNEL‐positive is red, and DAPI is blue. (B) Apoptosis index of each group; (C) The expression levels of Bcl‐2 mRNA were analysed in each group; (D) The expression levels of Bax mRNA were analysed in each group; (E) Protein bands of Bcl‐2 and Bax by western blot; (F) The protein expression levels of Bcl‐2 were evaluated for each group; (G) The protein expression levels of Bax were evaluated for each group. The measured data are expressed as mean ± SD. The experiment was repeated no less than three times. *p < 0.05 versus with MV12h group; **p < 0.01 versus with MV12h group; ***p < 0.001 versus with MV12h group; ****p < 0.0001 versus with MV12h group.

Prolonged MV can cause apoptosis of diaphragm cells, which can cause diaphragm damage leading to diaphragm dysfunction. Therefore, to explore the defensive impact of irisin on rat diaphragm tissue, we studied the apoptosis of cells in the diaphragm of rats. The MV12h group exhibited a noteworthy rise in apoptosis in the diaphragm tissue of rats, as indicated by the TUNEL staining portrayed in Figure 9A,B. And irisin effectively reduced the apoptosis of diaphragm cells (*p* < 0.05). Bax is a pro‐apoptotic gene in the Bcl‐2 gene family, and Bcl‐2 is an anti‐apoptotic gene. Downstream of ER stress activates apoptosis‐related genes, including Bcl‐2 and Bax, and eventually severe and sustained ER stress causes apoptosis. Matching with TUNEL staining, Bax expression was increased in the diaphragm of the MV12h group, and Bcl‐2 expression was decreased in the diaphragm of the MV12h group (*p* < 0.05). The administration of Irisin resulted in a decrease of Bax expression and an increase in the expression of Bcl‐2. (Figure 9C–G).

### 3.10 Irisin works by activating AMPK

**FIGURE 10:**
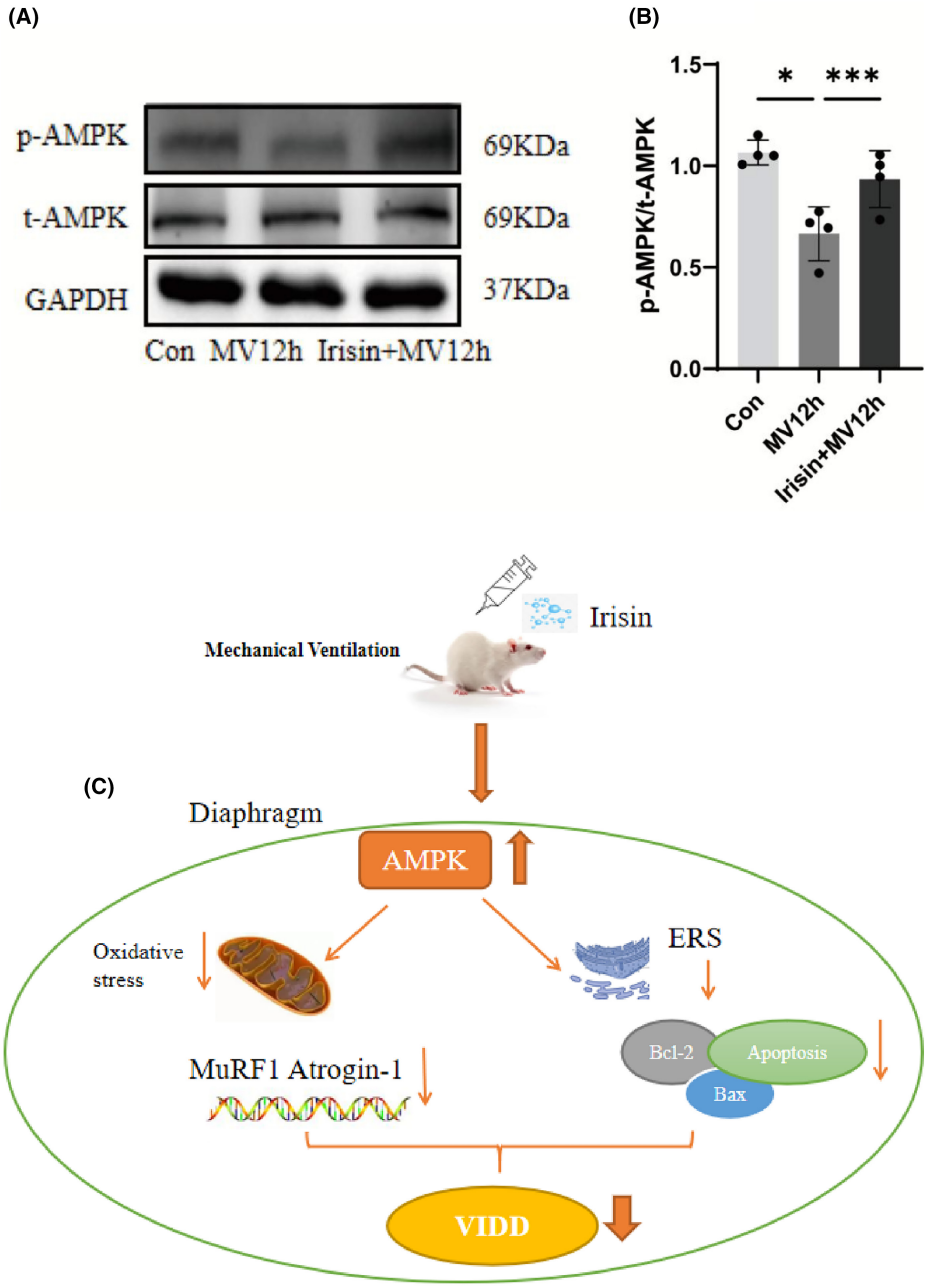
T‐AMPK and p‐AMPK protein expression in the diaphragm. (A) Protein bands of Bcl‐2 and Bax by western blot; (B) The p‐AMPK/t‐AMPK protein ratio of each group. (C) Technology roadmap for the article. The measured data are expressed as mean ± SD. The experiment was repeated no less than three times. *p < 0.05 versus with MV12h group; ***P < 0.001 versus with MV12h group. MV, mechanical ventilation; ER stress, endoplasmic reticulum stress; VIDD, ventilator‐induced diaphragmatic dysfunction.

The release of irisin from muscles relies on AMPK signalling, and irisin, in turn, has a self‐regulating impact that enhances the AMPK pathway, leading to an improvement in muscle function and metabolism. Our results showed that MV caused a decrease in phosphorylated AMPK expression in the diaphragm, and irisin reversed this change and upregulated phosphorylated AMPK expression, with a statistically significant difference (*p* < 0.05) (Figure 10).

## 4 DISCUSSION

Numerous studies have confirmed that long‐term MV causes VIDD. Our results confirmed that the expression of ER stress markers in the diaphragm increased with prolonged ventilation time during 24 h of mechanical ventilation, diaphragm dysfunction worsened, the expression of muscle atrophy‐associated proteins MuRF‐1 and Atrogin‐1/MAFbx increased, and the diaphragm CSA progressively became smaller with prolonged ventilation time. We found for the first time that irisin expression decreased with prolonged ventilation in rats. After administration of irisin, we found that diaphragm function and atrophy were improved in rats. Irisin downregulated the expression of ER stress markers and downstream levels of the pro‐apoptotic protein Bax. Irisin also reduced ROS and MDA levels and attenuated oxidative stress. AMPK was activated, and phosphorylation increased after irisin administration. Therefore, we speculate that irisin may reduce ER stress, oxidative stress and diaphragmatic apoptosis through activation of p‐AMPK, which may improve VIDD. This provides new ideas for clinical prevention and treatment of VIDD, and irisin may become a new target for the treatment of VIDD.

The VIDD in critically ill ICU patients has received a great deal of attention in the last decade and is strongly associated with weaning difficulties, hypoxemia and reintubation rates. However, we know very little about the mechanism and prevention measures of VIDD. Therefore, we constructed a VIDD model by mechanically ventilating rats to further study it. We found that diaphragm function was getting worse as the duration of MV increased. The main manifestations are a decrease in diaphragmatic contractility, a decrease in diaphragmatic resistance to fatigue, a decrease in the amplitude of the CMAP and a prolongation of its duration. The decrease in diaphragm contractility may be related to the presence of mitochondrial dysfunction, impaired ATP production and calcium ion disturbances in diaphragm cells during MV.^45^, ^46^ Atrophy of the slow muscle fibres of the diaphragm causes a decrease in fatigue resistance. The expression of atrophy‐related proteins MuRF‐1 and Atrogin‐1/MAFbx gradually increased. MuRF‐1 and Atrogin‐1/MAFbx are two E3 ligases that are mainly involved in the ubiquitin‐proteasome protein hydrolysis pathway. It was shown that ubiquitin‐proteasome‐mediated protein degradation is essential for diaphragm atrophy during MV.^47^ Diaphragm atrophy cannot be separated from a decrease in protein synthesis and an increase in protein catabolism, with protein degradation caused by the ubiquitin proteasome pathway being the main mode of muscle proteolysis. Consistent with this, HE staining of the diaphragm also showed a decrease in muscle fibre CSA with increasing ventilation time.

Recently, ER stress has been reported to cause diaphragm atrophy and contractile dysfunction in septic rats.^32^ Previous studies have shown that sepsis‐related diaphragm dysfunction and VIDD share many key molecular mechanisms, such as oxidative stress, cytokine overexpression and mitochondrial dysfunction.^33^ A recent study reported that ER stress may be a cause of VIDD. ER stress can cause diaphragm atrophy and weakness.^25^ Interestingly, our study found a progressive increase in the expression of ER stress markers in VIDD. In general, cells regulate the protein folding capacity of the endoplasmic reticulum according to their needs. Appropriate level of ER stress can regulate the metabolism and formation of skeletal muscle by maintaining Ca^2+^ balance and promoting protein folding to improve muscle contraction function.^12^ Prolonged MV causes an increase in oxidative stress and calcium ion disorders, which are activators of ER stress. Thus, ER stress is activated in VIDD. Our results showed that the expression of ER stress markers GRP78, CHOP and GRP94 gradually increased with prolonged ventilation time. Chronic ERS‐induced unfolded protein responses have been shown to be involved in activation of protein hydrolysis pathways, inhibition of protein synthesis and regulation of skeletal muscle mass and metabolic function.^18^ The downstream of ER stress is closely related to apoptosis, which mainly includes the activation of apoptotic proteins such as caspase family and Bax. The apoptotic pathway may also contribute to diaphragmatic dysfunction.^48^

Irisin, a novel muscle factor, has shown benefit in many muscle diseases. In the present study, it was found for the first time that irisin expression in the diaphragm was progressively reduced with increasing ventilation time. Serum levels of irisin also decreased accordingly. This may be clinically useful in assessing the severity of VIDD and in predicting time off the ventilator. Our data show for the first time that irisin pretreatment reduced the levels of GRP78, CHOP and GRP94 during VIDD, thereby reducing MV‐induced ER stress. ER stress activates caspase‐12 and CHOP‐dependent apoptotic pathways, affecting Bax/Bcl‐2 expression and caspase‐3 activation.^17^, ^49^, ^50^ Our results showed that MV resulted in the increased apoptotic index, elevated Bax expression and decreased Bcl‐2 expression in the diaphragm. Irisin pretreatment attenuated diaphragm apoptosis, down‐regulated Bax expression and up‐regulated Bcl‐2 expression levels. Recent studies have reported that ER stress activates oxidative stress and that a vicious cycle between the two also exacerbates the development of VIDD.^25^ When the protein folding capacity of the endoplasmic reticulum is overwhelmed, the endoplasmic reticulum releases Ca^2+^, causing mitochondrial calcium overload, which leads to pathological changes in the mitochondrial membrane. Our data suggest that MV leads to increased release of ROS in the diaphragm and increased production of the lipid peroxidation marker MDA. In contrast, irisin increased the activity of the antioxidant enzyme GSH‐Px, decreased diaphragm MDA levels and inhibited ROS production. Irisin can enhance the antioxidant enzyme activities of superoxide dismutase (SOD) and GSH‐Px in skeletal muscle.^38^ In summary, irisin can reduce ER stress and the resulting CHOP pathway‐related apoptosis and oxidative stress to improve diaphragm function.

Numerous studies have found that the beneficial effects of irisin are achieved through regulation of the AMPK pathway. AMPK, a major energy metabolism‐regulating kinase, controls system energy balance and metabolism.^51^ Activation of AMPK can act as a physiological inhibitor of ERS and has important clinical implications for the development of therapeutic strategies for VIDD. Irisin has recently been reported to protect cardiomyocytes from hypoxia/reoxygenation injury by attenuating AMPK‐mediated ERS.^28^ AMPK is a serine kinase with a wide range of biological roles. Stressors such as inflammation, hypoxia and oxidative stress can promote AMPK phosphorylation and compensate for its anti‐inflammatory and anti‐ER stress effects.^52^, ^53^, ^54^ Our data suggest that irisin increases the level of phosphorylated AMPK. Whether irisin activates AMPK directly or through some intermediate receptor has not been determined. More in‐depth studies are needed specifically on how AMPK regulates ER stress.

However, there are many limitations to this study. Firstly, in selecting the study subjects, we chose healthy SD male rats. We chose rats as an experimental model because rat and human diaphragm have similar anatomical features, functional characteristics, fibre type composition and show parallel time course in the development of VIDD.^55^, ^56^ We did not include female rats because studies have shown that there are no sex differences between male and female rats in the development of VIDD.^57^, ^58^ Secondly, critically ill patients requiring MV in clinical settings often have a combination of diseases (e.g. sepsis, multiple organ dysfunction, etc.), and we chose healthy animals without considering the effects of other factors on the diaphragm. In future studies, more MV models that match the pathological characteristics of critically ill patients need to be included. Finally, more research is needed on the specific mechanisms and details of how irisin regulates AMPK.

## 5 CONCLUSION

In conclusion, our study mainly found that ER stress in the diaphragm gradually increased with prolonged ventilation time, and diaphragm dysfunction gradually worsened. Irisin could protect the diaphragm by reducing ER stress by upregulating AMPK pathway and reducing diaphragm apoptosis and oxidative stress. ER stress, as one of the causative factors of VIDD, plays many roles in the development of VIDD. Targeted AMPK and irisin administration may hold promise for the prevention and treatment of VIDD, although further studies are needed for clinical application. The above findings provide insights into the formation of VIDD, which may have therapeutic implications for both the prevention and treatment of VIDD as well as potential drug targets.

## AUTHOR CONTRIBUTIONS

**Jumei Zhang:** Conceptualization (lead); data curation (lead); formal analysis (lead); investigation (equal); methodology (equal); project administration (lead); resources (equal); software (equal); supervision (equal); validation (equal); visualization (equal); writing – original draft (lead); writing – review and editing (lead). **Rui Tu:** Formal analysis (equal); methodology (equal); project administration (equal); software (equal); validation (equal); visualization (equal); writing – original draft (equal). **Fasheng Guan:** Formal analysis (equal); investigation (equal); methodology (equal); resources (equal); software (equal). **Jianguo Feng:** Formal analysis (equal); investigation (equal); methodology (equal); project administration (equal). **Jing Jia:** Conceptualization (equal); investigation (equal); resources (equal); software (equal); supervision (equal). **Jun Zhou:** Methodology (equal); project administration (equal); resources (equal); supervision (equal); writing – original draft (equal). **Xiaobin Wang:** Investigation (equal); methodology (equal); project administration (equal); resources (equal). **Li Liu:** Conceptualization (equal); data curation (equal); funding acquisition (equal); project administration (equal); resources (equal); software (equal); visualization (equal); writing – review and editing (equal).

## CONFLICT OF INTEREST STATEMENT

The authors declare that there are no conflicts of interest.

## Data Availability

The data used to support the findings of this study are available from the corresponding author upon request.
