# Correction to ‘Irisin Attenuates Ventilator‐Induced Diaphragmatic Dysfunction by Inhibiting Endoplasmic Reticulum Stress Through Activation of AMPK’

**DOI:** 10.1111/jcmm.71260

**Published:** 2026-07-28

Zhang, J. , Tu, R. , Guan, F. , Feng, J. , Jia, J. , Zhou, J. , Wang, X. , and Liu, L. . “Irisin Attenuates Ventilator‐Induced Diaphragmatic Dysfunction by Inhibiting Endoplasmic Reticulum Stress Through Activation of AMPK,” Journal of Cellular and Molecular Medicine 28, no. 9 (2024): e18259, 10.1111/jcmm.18259.38676364 PMC11053354

In Ju Zhang et al. [1], the images of the Con group and the Irisin + MV12h group in Figure 6G overlapped due to a technical error during image preparation. The correct figure is shown below. The authors confirm all results and conclusions of this article remain unchanged.

**FIGURE 6:**
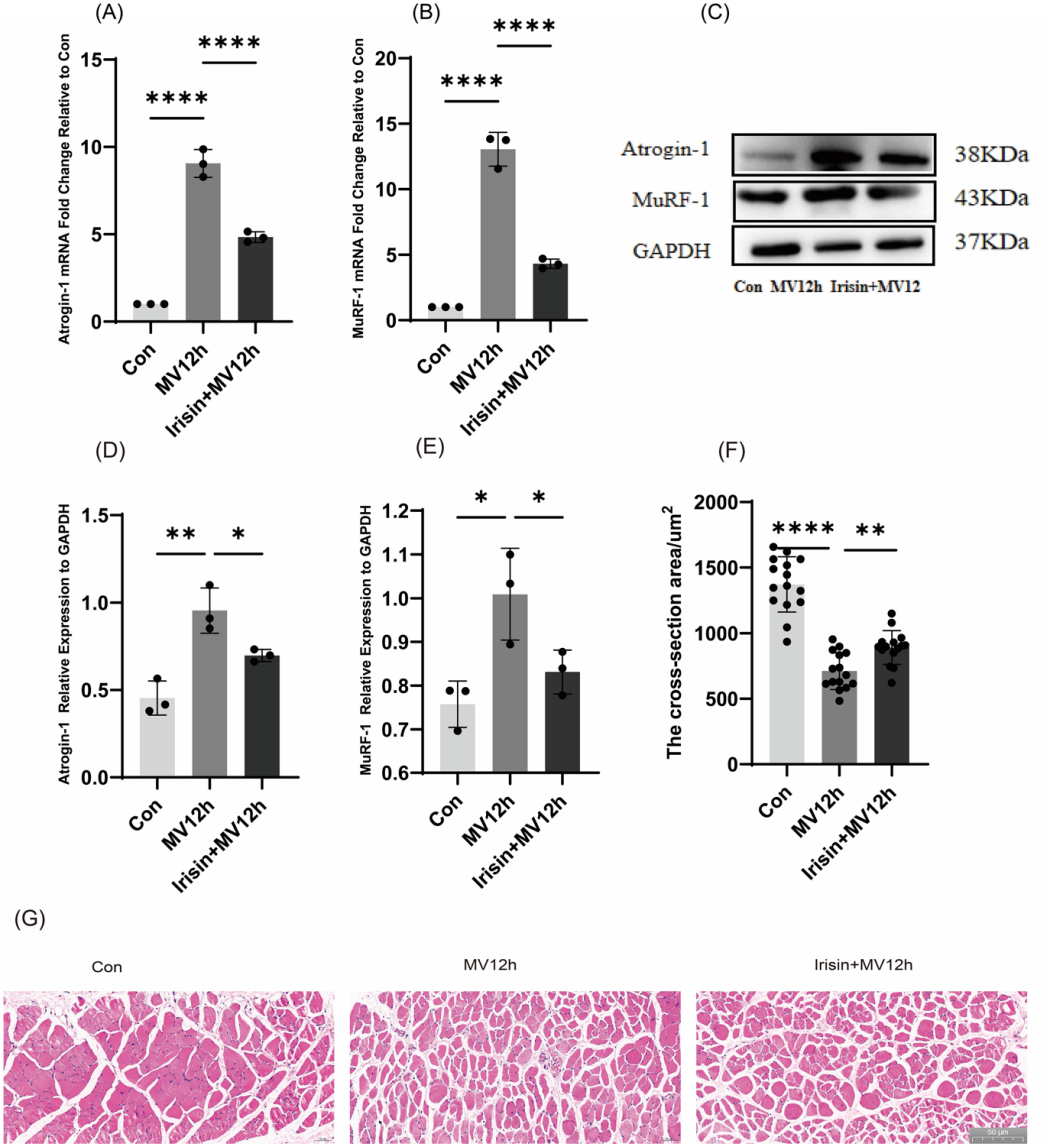
Effect of irisin on diaphragmatic atrophy in MV. (A) The mRNA expression levels of Atrogin‐1/MAFbx in each group; (B) The mRNA expression levels of MuRF‐1 in each group; (C) Protein bands of MuRF‐1 and Atrogin‐1/MAFbx by western blot; (D) The protein expression levels of Atrogin‐1/MAFbx were evaluated for each group; (E) The protein expression levels of MuRF‐1 were evaluated for each group; (F) Results of diaphragm cross‐sectional area statistics; (G) Representative images of each group of H&E staining. The measured data are expressed as mean ± SD. The experiment was repeated not less than thrice. *p < 0.05 versus with MV12h group; **p < 0.01 versus with MV12h group; ****p < 0.0001 versus with MV12h group. MV, mechanical ventilation.
